# Achieving Sustainability and Scale-Up of Mobile Health Noncommunicable Disease Interventions in Sub-Saharan Africa: Views of Policy Makers in Ghana

**DOI:** 10.2196/11497

**Published:** 2019-05-03

**Authors:** Daniel Opoku, Reinhard Busse, Wilm Quentin

**Affiliations:** 1 Faculty VII Economics and Management Institute of Technology and Management Department of Health Care Management Technische Universität Berlin Berlin Germany

**Keywords:** implementation science, mHealth, eHealth, noncommunicable diseases, disease management, sub-Saharan Africa, qualitative research, health policy

## Abstract

**Background:**

A growing body of evidence shows that mobile health (mHealth) interventions may improve treatment and care for the rapidly rising number of patients with noncommunicable diseases (NCDs) in sub-Saharan Africa (SSA). A recent realist review developed a framework highlighting the influence of context factors, including predisposing characteristics, needs, and enabling resources (PNE), for the long-term success of mHealth interventions. The views of policy makers will ultimately determine implementation and scale-up of mHealth interventions in SSA. However, their views about necessary conditions for sustainability and scale-up remain unexplored.

**Objective:**

This study aimed to understand the views of policy makers in Ghana with regard to the most important factors for successful implementation, sustainability, and scale-up of mHealth NCD interventions.

**Methods:**

Members of the technical working group responsible for Ghana’s national NCD policy were interviewed about their knowledge of and attitude toward mHealth and about the most important factors contributing to long-term intervention success. Using qualitative methods and applying a qualitative content analysis approach, answers were categorized according to the PNE framework.

**Results:**

A total of 19 policy makers were contacted and 13 were interviewed. Interviewees had long-standing work experience of an average of 26 years and were actively involved in health policy making in Ghana. They were well-informed about the potential of mHealth, and they strongly supported mHealth expansion in the country. Guided by the PNE framework’s categories, the policy makers ascertained which critical factors would support the successful implementation of mHealth interventions in Ghana. The policy makers mentioned many factors described in the literature as important for mHealth implementation, sustainability, and scale-up, but they focused more on enabling resources than on predisposing characteristics and need. Furthermore, they mentioned several factors that have been rather unexplored in the literature.

**Conclusions:**

The study shows that the PNE framework is useful to guide policy makers toward a more systematic assessment of context factors that support intervention implementation, sustainability, and scale-up. Furthermore, the framework was refined by adding additional factors. Policy makers may benefit from using the PNE framework at the various stages of mHealth implementation. Researchers may (and should) use the framework when investigating reasons for success (or failure) of interventions.

## Introduction

### Background

With the drastic decline of communicable, maternal, and neonatal diseases as cause of death and burden of disease across the globe [1], and particularly in Africa [1,2], the epidemiologic transition toward noncommunicable diseases (NCDs) is in full swing. By 2030, 42% of all projected deaths in sub-Saharan Africa (SSA) will be caused by NCDs, which will then surpass communicable diseases as the leading cause of death in the subregion [3,4]. In some African countries such as Ghana, statistics show that already today about 42% of the total annual deaths are caused by NCDs, led by cardiovascular diseases [5-7].

At the same time, most countries in SSA have become eager adopters and innovators of the use of mobile and digital technologies. In Ghana, as early as 2013, “[m]ore than four out of every five households (80.3%) in the country own[ed] a mobile phone” [8], thereby expanding the opportunities for the implementation of mobile phone–based health (mHealth) interventions [9,10]. Numerous studies and reviews have reported positive results of mHealth interventions against NCDs [11-15]. The World Health Organization (WHO) promotes the further development and more widespread use of mHealth interventions as part of its Global Action Plan for the prevention and control of NCDs [16]. Nevertheless, most mHealth interventions remain at the stage of pilot projects, and they are almost never scaled-up to entire countries [10,13,17-19].

Efforts at international and European levels have aimed to provide guidance to countries to support scale-up of mHealth interventions and integration into routine care practices [20-22]. For example, WHO and the International Telecommunication Union have produced a detailed toolkit to support the development of national electronic health (eHealth) strategies [21]. The toolkit is focused on the role of enabling legislation and regulation, government and sector buy-in, and planning and funding for implementation and sustainability. More recently, the European Union–funded Momentum project for successful implementation of telemedicine into routine health care has published a list of 18 factors to make telemedicine a success, which also include legislation and sector buy-in, but further recommend consideration of the cultural readiness toward telemedicine and the identification of a compelling need [22].

**Figure 1:**
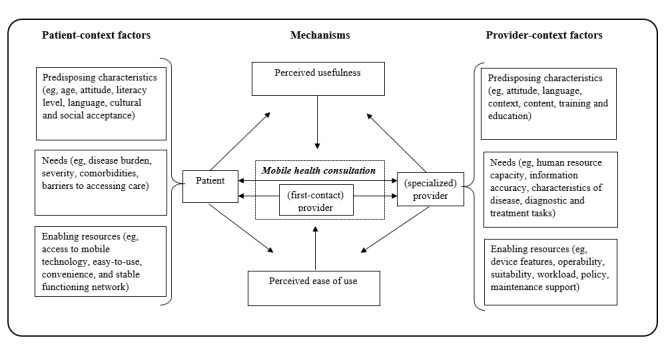
Mobile heatlh predisposing characteristics, needs, and enabling resources framework.

In a recent realist review, Opoku et al [23] developed a theoretical framework that aims to provide guidance to policy makers and other decision makers working on implementing, sustaining, and scaling-up mHealth interventions for NCD management in SSA. The framework hypothesizes that “predisposing characteristics and need of patients and healthcare providers as well as the availability of enabling resources in the community influence the perceptions of patients and providers that mHealth interventions are useful and easy to use—and these perceptions are essential for the successful implementation of an mHealth intervention” [23]. As shown in Figure 1, the framework focuses attention on the influence of context factors, including predisposing characteristics, needs, and enabling resources (PNE), for the long-term success of mHealth interventions. Therefore, we use the term *mHealth* *PNE framework* for the rest of the paper.

The mHealth PNE framework is grounded in the experiences of patients and health care providers as reported in 20 studies of 18 mHealth interventions for NCDs [14,24-42] conducted in 10 SSA countries. It combines the Andersen behavioral model of health services utilization with the Davis technology acceptance model [43,44]. The framework focuses attention on a large set of—yet to be further refined—contextual factors that can be grouped under PNE. For example, cultural readiness mentioned as one of the 18 factors of the momentum group would fall under *predisposing characteristics*, whereas establishment of an appropriate legal environment would fall under *enabling resources* and identification of patients’ needs under *needs*. However, it remains unknown whether the context factors that have so far been identified under the categories of PNE are in line with the views of policy makers and other decision makers about the most important conditions for implementation, sustainability, and scale-up.

Ghana is one of the countries in SSA where efforts to support the development of mHealth interventions have been most pronounced [45-48]. These efforts include the development of the Ghana eHealth Strategy, which aims at supporting the improvement of the overall performance of the health sector [47]. In addition, several mHealth interventions have been implemented, including the Millennium Villages telemedicine project in the Amansie West district [49] and the Mobile Technology for Community Health program in 7 districts [50,51]. As a result, policy makers in Ghana can be expected to have considerable experience with mHealth interventions, and they are likely to have thought about factors that support intervention sustainability and scale-up.

The views of policy makers and other decision makers will ultimately determine the implementation, sustainability, and scale-up of mHealth interventions in SSA. To assure that the mHealth PNE framework is useful as a guide for policy makers, it is important that the framework is sufficiently aligned with their thinking, that is, policy makers should find the categories of the framework useful when considering the most important factors for implementation, sustainability, and scale-up. In addition, the experiences of policy makers may provide additional insights about the important factors contributing to a successful implementation, sustainability, and scale-up of mHealth NCD interventions that might be missing in the existing literature [23].

### Objectives

Therefore, the aim of this study was to understand the views of policy makers with regard to the most important factors that should be considered to assure successful implementation, sustainability, and scale-up of mHealth NCD interventions, thus contributing to the improvement of the mHealth PNE framework. More specifically, the study sought to (1) assess policy makers’ knowledge of and attitude toward mHealth NCD interventions, (2) identify whether the categories of the framework are useful to structure the thinking of policy makers, and (3) integrate the perspectives of policy makers into the various components of the framework.

## Methods

Application for ethical review of the study was submitted to the Committee on Human Research, Publications, and Ethics at the Kwame Nkrumah University of Science and Technology, School of Medical Sciences, and Komfo Anokye Teaching Hospital, Kumasi, Ghana, and final approval was received on February 25, 2016. The study was conducted using qualitative methods (interviews) and by applying a qualitative content analysis (QCA) approach [52]. The paper was drafted following the Consolidated Criteria for Reporting Qualitative Studies [53].

### Qualitative Interviews

Informed consent was first sought and participants were given sufficient information, including about the risks and benefits of participating in the study. Guided by a semistructured questionnaire (Multimedia Appendix 1), qualitative interviews were conducted by DO between November 2015 and January 2016 among stakeholders at the health policy direction level, who were actively involved in national health policy decision making and implementation in Ghana. These one-to-one interviews lasted for an average of 45 min and were recorded for transcription and analysis. In addition, field notes were taken for purposes such as capturing off-tape records and explaining why an interview might have been poorly conducted.

### Participants

Participants were from diverse backgrounds and generally worked at high levels of hierarchy and responsibility in different institutions. They had a long-standing experience working in various sectors of the Ghana national health system, particularly on NCDs and other related subjects. All participants were involved in drafting and developing the 2012 National Policy for the Prevention and Control of Chronic Non-Communicable Diseases in Ghana. As such, all participants had been involved in defining the technical direction and framework for implementing NCD-related programs in the country [54].

### Selection Criteria

A list of the members of the technical working group for Ghana’s national NCD policy was retrieved from the document titled *Strategy for the Management, Prevention and Control of Non-Communicable Diseases in Ghana* by the Republic of Ghana, Ministry of Health [54]. The list consisted of a total number of 19 members who were contacted by DO and who received information about the study via emails, telephone calls, and Skype calls. They were medical doctors, including general practitioners and public health consultants, epidemiologists, political scientists, (public health) lecturers, public health researchers, health educators, program managers, disease surveillance officers, international health specialists, program coordinators, (public health) pharmacists, dieticians, public health practitioners, policy advisors or analysts, planning officers, and freelance nutritionists. Participation was voluntary, and participants were assured that information such as names and addresses that could lead to their identification would be avoided to ensure privacy. Those who responded were followed up for the interviews. No restrictions were imposed except that the participation was based on the availability and willingness to contribute during the period of data collection.

### Analysis

**Figure 2:**
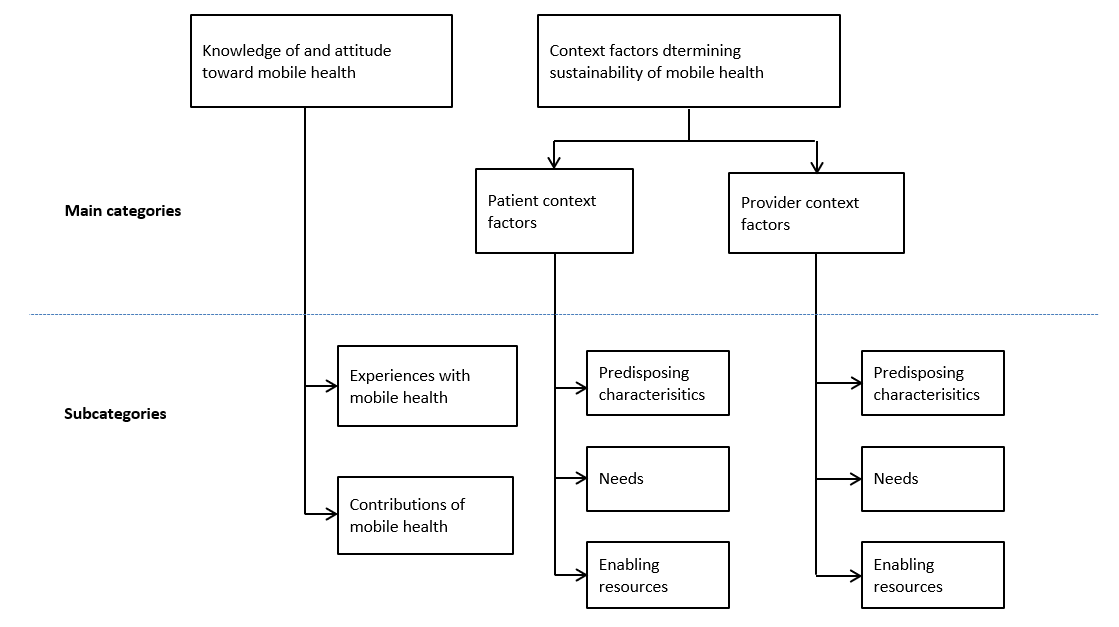
Coding frame for analysis based on the mobile health predisposing characteristics, needs, and enabling resources framework.

Following the QCA approach [52,55], the coding frame in Figure 2 was used for the analysis. It was largely based on the mHealth PNE framework. The framework theorizes that successful implementation of mHealth interventions is determined by context factors— *predisposing characteristics, enabling factors, and needs* —of patients and health care providers, which influence their perceptions on the usefulness and ease of use of the intervention [23]. Thus, for example, whether a mobile phone–based self-monitoring blood glucose intervention designed for diabetes care in Ghana will be successful or not depends on whether both diabetic patients and their health care providers perceive the intervention to be useful and easy to use.

According to the framework, the perceived usefulness and ease of use of an intervention are determined by (1) patients’ predisposing characteristics, such as age, attitude, literacy, language, and cultural or social acceptability; (2) their need, such as reducing financial burden of care and avoiding long travel or waiting time; and (3) the factors that will enable them to utilize the intervention well, which may include access to a mobile phone and a stable network [23]. In addition, perceived usefulness and ease of use of providers depend on predisposing characteristics (eg, technology-related training), needs (eg, human resource capacity), and enabling resources (eg, tolerable workload and incentives) [23].

The interview transcripts for the analysis were first coded by DO and subsequently reviewed by both DO and WQ, according to the various components of the framework and grouped into main categories and subcategories. The 2 main categories were *knowledge of and attitudes toward mHealth* and *context factors determining sustainability of mHealth (patient-context factors* and *provider-context factors).* The results were analyzed mainly based on the 3 subcategories of the framework (ie, *predisposing characteristics, needs, and enabling resources*) and then presented thematically under each of the main categories. The analysis also sought to identify other potentially relevant factors missing in this framework.

## Results

### Characteristics of Participants

Out of the 19 policy makers who were contacted, 13 participated in the study. The participant policy makers had long-standing experiences with an average of 26 years in managing various health programs, interventions, and departments and were actively involved in health policy making processes in Ghana. With the exception of 4 participants who had retired at the time the interviews were conducted, all were serving in high-level (national) capacities at the Ministry of Health, Ghana Health Services, academia, and the public and private sectors. Table 1 summarizes the characteristics of the participants.

**Table 1:** Characteristics of the participant health policy makers and managers in Ghana.

Gender^a^	Age (years)	Working experience with noncommunicable diseases	Experience (years)
Male	>50	Medical practice, program management, policy	21
Male	>50 (retired)	Medical practice, program management, policy	36
Male	>50 (*retired*)	Medical practice, teaching, research, program management, policy	41
Male	>50	Research, health information management, policy	33
Male	>50	Health education, training, research, communication, program management, policy	25
Male	40-44	Health regulations, disease control and prevention, policy	19
Male	40-44	Medical practice, disease control and prevention, policy	13
Female	45-49	Clinical practice, health promotion, policy	20
Male	45-49	Teaching, research, consultancy	16
Female	>50 (retired)	Health promotion, disease prevention, policy	>30
Male	>50	Health sector coordination, program management, policy	30
Male	>50	Teaching, research, consultancy	21
Female	>50 (retired)	Health promotion, advocacy, policy	34

^a^Source: authors’ own compilation.

**Table 2:** Identified beneficial applications of mobile health interventions.

Health promotion and prevention^a^	Education and awareness creation (R^b^: 1, 5, 7, 10), follow-up (R: 1), information centers (R: 11), interactive platform (R: 12)
Health care delivery (maternal and child health care)	Scheduling/adherence/compliance/reminder (appointment and medication) (R: 1, 2, 3, 7, 11, 12), (emergency/specialist) referrals (R: 2, 3, 13), follow-up (R: 1, 11), community-based health care (R: 4, 11), digitalized hospital records (R: 6, 8), record-keeping (vital statistics) (R: 4), creating access to health care (R: 4), health information and follow-up (for pregnant women) (R: 13)
Noncommunicable disease–related management	Education (regenerative health and nutrition) (R: 4, 8, 11), specialized care (for complicated cases) (R: 9, 12), appointments/reminders for testing fasting blood sugar (R: 3), checking/monitoring vital signs (blood pressure and retina check) (R: 4), control and prevention of hypertension (awareness creation, and reminders for drug refill) (R: 7), early detection of complications [R: 9], cancer registry (R: 11), follow-ups (R: 11)

^a^Source: authors’ own compilation.

^b^R stands for respondent and the following numbers assigned in this study.

### Knowledge of and Attitude Toward Mobile Health Interventions

Interviewed policy makers had considerable knowledge of mHealth interventions, broadly in relation to the general field of health (prevention and health promotion and health care delivery—maternal and child health care) and some specifically relating to NCDs (hypertension prevention and control). Table 2 presents the beneficial applications of mHealth interventions, sorted out according to the highest number of participants who identified them. Some of the policy makers actually had a long-time experience with mHealth and had been involved in the use of mobile phones to support health care delivery, either as providers or as patients themselves.

All interviewees agreed that mHealth interventions can contribute to improved NCD management in Ghana. They identified a range of potentially beneficial applications: awareness creation and (regenerative) health education, early detection of NCD conditions, reduction of waiting time, follow-ups and monitoring, keeping track of the appointments of patients, vital statistics and adherence to medication, emergency alert, creating registries, record keeping, dissemination of evidence, and ensuring sustainable health care. Most importantly, the policy makers highlighted that mHealth could potentially help patients to better manage their NCDs and improve treatment compliance:

[it] can keep patients in care, reduce morbidity, reduce mortality. Definitely because it's all a matter of keeping them [ie, patients] in care and ensuring that they learn the good practices and all that. So, I think it would help the outcomes; we will get better outcomes, reduce the disabilities from NCDs, and also reduce the mortalities from NCDs, definitely!

Respondent: 1

For me the biggest impact is that it will help to manage treatments, it will reduce treatment failures, and it will help people to be more productive so that people can then take better care of themselves and not spend all the time going to the hospitals.

Respondent: 4

At the same time, interviewees noted that mHealth interventions provide a solution for only some of the problems of NCD management in Ghana:

I always get worried when people try to use technology as a “fix all”. Technology is not a fix all, it fixes some problems but not all problems and it’s contextual.

Respondent: 1

Given the complexity of NCD management, mHealth consultations were considered to be safe and suitable mostly for follow-ups, after an initial contact between patients and providers has been established:

...there are huge potentials when it comes to the use of mobile phones but for noncommunicable diseases, the evidence is not very clear for us...from our experience it has to be a “postcontact” intervention. There is always the first contact that has to be made [at the facility] and thenthe intervention kicks in as a follow-up,only after the initial contact. If you don’t have initial contact with the hospital, the opportunity to rope in ICT to help you to readjust and to be healthier becomes a bit of a problem.

Respondent: 4

However, the policy makers maintained that the use of mobile phones in health care is becoming an important strategy in Ghana, particularly in reducing maternal mortalities and controlling epidemics. In fact, the policy makers were enthusiastic about the potential of mHealth to improve NCD management in Ghana:

It is a very good idea, brilliant idea! I mean it is something that we’ve always been talking about that people should be able to stay in their houses and manage or even call doctors to come or even call for advice from doctors.

Respondent: 5

### Perspectives of Policy Makers on Context Factors Determining Sustainability of Mobile Health in Ghana

This section presents the interview results categorized along the 3 context factors of the analytical framework, that is, *predisposing characteristics, needs, and enabling resources*. Table 3 provides a summary of the identified factors supporting and/or expanding the framework, arranged in a descending order of the most frequently mentioned factors by the participants.

#### Predisposing Characteristics

According to the framework, the most important predisposing characteristics supporting the implementation of mHealth interventions are a positive attitude, cultural/social acceptance, and a common language of communication. The interviewed policy makers, however, identified age, literacy, and level of education, as well as providers’ continuous training, upgrade, and education as more important factors:

The youth are very good at these things and so if you work it out with them it would work. The problem is, are the youth the people who actually go for the services? And so, the majority of the people who would be having noncommunicable diseases are not the youth and they are the people who would not understand this.

Respondent: 3

My fear is the illiteracy rate. How many people with mobile phones know how to send a text message? How many people can even store or delete numbers or messages? They don’t know, they have the phones for receiving calls and making calls, that’s all!

Respondent: 2

We need to do more in terms of training our providers. We call it the two ends, or supply and demand. That is, we supply and the population would demand. So, let us tailor a kind of training for the suppliers of the services in terms of the use of mobile phones and then also teach some of our clients on the use of the mobile phone in terms of getting access to some of these specialists, because I still want to believe that the management of NCDs is a specialized service, which cannot be left in the hands of ‘ordinaries’.

Respondent: 11

They also stressed that, more generally, trust and confidence among health care providers is a prerequisite for successful implementation of mHealth:

The other thing would have to do with the [health] staff attitude. The major question is that, for example, if I [a specialized health care provider] at Korle-Bu teaching hospital [in Accra] give instructions to those [health care providers] in the rural areas, how sure am I that they are doing what I am instructing them to do? And if anything at all should go wrong, who is to be blamed, me or those out there? So, amongst the health staff, there are usually pessimistic views and so some of them will not be interested in these innovations but others might.

Respondent: 9

In addition, interviewees highlighted that attitudes of patients related to myths, misconceptions, fear of change, and phobia for technological innovations may negatively impact patients’ perceptions about the usefulness of mHealth interventions:

We need to look at people’s phobia for technology and see how that barrier can be broken.

Respondent: 6

Maybe we still maintain the old ways of doing things; we don’t like change. Africans in general, but Ghanaians especially, we fear change. It is a fact that we fear about what if it doesn’t work out well and who takes the fall for it!

Respondent: 10

With regard to predisposing characteristics influencing the perceived ease of use, interviewees believed that urban populations are more familiar with mobile technologies. However, in general, the Ghanaian population was thought to be ready to use mobile phones for health care given the high penetration of the technology.

**Table 3:** Summary of the identified factors supporting the mobile health predisposing characteristics, need, and enabling resources framework.

Mechanism context^a^	Patient		First contact/specialized provider	
	Perceived usefulness	Perceived ease of use	Perceived usefulness	Perceived ease of use
Predisposing characteristics	(Local) language [R^b^:5,6,11,12]; myths, fear/phobia, misconceptions [R:2,5,6,10];*informed, convinced, trust, and confidence (satisfaction)* [R:2,8,11]; *locality (urban/rural)*^c^ [R:2,8]; socioculture [R:4,7]; acceptance [R:5,6]; (positive) attitude [R:5,12]; self-motivation [R:3]; age [R:8]; *gender* [R:8]; social class (middle) [R:1]	Literacy and level of education [R:1,2,3,4,5,6,7,11,12]; age (youth ≥10 years, adults) [R:2,3,5,7,10,13]; penetration, and familiarity (urban) [R:1,5,6,13]; training, know-how, confidence [R:3,4,5,12]; basic, simple [R:6,8]; *personalization* [R:8,11]	(Positive) attitude interest, dedication, willingness, and motivation [R:1,8,12]; *good (provider-patient/community)* relationship [R:4,8,11]; language [R:5,9]; trust and confidence [R:11]; ready to support [R:13]	Continuous training, upgrade, and education [R:4,7,9,10,11,12]
Need	Health care access barriers (poverty, transportation, ineffective health facilities, distance, travel and waiting time, cost, urgency and quality of care, stress reduction, and satisfaction) [R:2,3,4,9,10,12,13]; disease condition (severity, upsurge, uncertainties of care) [R:1,2,4,6,9,13]; *need for urgent/special care* [R:7,8,9,13]	*Technology-driven* *need/demand* [R:2,3,4,6,13]	Reduce burden of cases/workload [R:2,6,10,11,12,13]; lack of human resources (limited specialists, unequal distributions of professionals, lack of motivation) [R:9,11,12,13]; *integrated care* [R:3,10,13]; lack of necessary systems and infrastructure (health facility, referral system, transport) [R:9,11]; *continuity of care* [R:1,13]; lack of accurate information [2,11]; reduce morbidity/ mortality [R:11,12]; *exchange of expertise* [R:9]; cost-saving [R:9]; enhance emergency care [R:11]	Characteristics of disease, diagnostic and treatment tasks (stage) [R:4,9,11,12]; information need [R:2,10]
Enabling resources	Functioning infrastructure (mobile network/connectivity, transport system, electricity, basic test equipment) [R: 1,4,6,7,8,9,11,12,13]; access to mobile phone [R:1,4,6,7,8,11,12,13]; availability and affordability of (telecommunication) services [R:1,3,5,6,11,12,13]; *partnership and support* [R:2,3,7,9]; awareness creation [R:2,5]; *avoidance of abuse* [R:4,12]; convenience [R:6]; confidentiality and privacy [R:8]; (community) support [R:10]	Portability and easy to use [R:6,13]; *(family) support* [R:8]; maintenance (battery recharge) [R:12]	Legislation and policy (phone usage, liability, funding mechanisms and reimbursement, data security and privacy, staff job description, partners) [R:1,2,4,5,6,7,8,9,13]; *(government, institutional, sectoral, stakeholders’) support)* [R:1,4,5,7,9,10,12,13]; infrastructure (functioning network services, equipment) [R:1,5,6,8,10,11]; financial resources and incentives [R:1,6,9,10,11,12]; quality, availability and affordability of services [R:1,7,10,12]; sustainability plan [R:7,10,12,13]; phone access [R:1,4,10]; *documentation and record-keeping* [R:1,2,9]; cost-effectiveness [R:5,8,10]; *evidence-informed (research, expert advice)* [R:5,10,11]; awareness [R:10]; (mobile health) guidelines [R:1]; *abuse/corruption* [R:11]	Simple, safest and easy technologies/ intervention (apps and softwares) [R:1,4]; type of (available) technologies [R:1]; maintenance [R:6]; phone features (screen, tailored operability) [R:7]

^a^Source: authors’ own compilation based on interview results.

^b^R indicates the reference citations.

^c^Text in italics are the additional patient- and provider-context factors of the mobile health PNE framework identified in this study.

#### Needs

The framework stipulates that patient and provider needs, such as access barriers for patients (eg, long travel times and costs) and providers’ lack of capacity to provide adequate care, influence the utilization of mHealth interventions in SSA. In this study, the interviewed policy makers suggested that patients who face health care access barriers of various forms and nature are more likely to perceive mHealth interventions as useful (see Table 3). They considered patients with severe conditions and/or in need of special/urgent care to benefit most from mHealth interventions, particularly if the interventions contribute to reduced travel times and better access to providers:

It would be useful; it would reduce a whole lot of travelling time and reduce some stress levels in getting vehicle/transport. It may even cut down on mortality because it can enhance emergency treatment and emergency care.

Respondent: 11

It would be very much useful in our settings and circumstances where many people do not even have access to the health facilities because of absence of the health facility, low numbers of health workers; that is, the low patient to health worker ratio. If health professionals can be reached via mobile phones or other ICTs, that would improve the chances of more people getting access and it would even lead to realizing the universal health coverage.

Respondent: 12

Once we are able to do this, we would save more lives and then again we would have lesser cases developing into complications to demand more attention and more time from the experts.”

Respondent: 9

Furthermore, interviewees mentioned several tasks for which mHealth interventions would respond to the needs of health care providers, thus contributing to perceived usefulness and ease of use of mHealth interventions. For example, to reduce the workload on providers and to use mobile phones for regular monitoring of blood sugar levels of diabetic patients:

We are aware that the health system in Ghana is stricken by lack of facilities and diagnostics, and the health staffs are not motivated to go and stay in the rural areas...and because we won’t have enough doctors and enough experts in the rural areas then we can’t run away from telemedicine.

Respondent: 9

...if we are talking specifically about testing fasting blood sugar, it shouldn’t be that the patients wait at the clinic...because we all know that the patients have to fast and for a diabetic, once you haven’t taken the blood sample s/he cannot eat. There should be enough health care providers available at all times to attend to them immediately. And so that should be organized well, a mobile phone can help do that easily...

Respondent: 3

I think it is time we do it, it would even reduce the workload on me [the provider].

Respondent: 2

Notably, the framework did not specify which particular needs of patients influence their perceived ease of use of mHealth interventions. Nonetheless, the interviewed policy makers suggested that the general trend to use information technology for other services may create a need to use mHealth in the management of NCDs, while simultaneously making it easier to use the technology:

We are in a technology age; whether we like it or not, technology is taking over and the earlier we get ourselves involved the better, because there would be a time where all banking would be done online. So, the fact that one is not computer literate nor mobile phone literate it cannot be assumed that the world should wait for us. So, it has to be done and it is being done.

Respondent: 3

#### Enabling Resources

Enabling resources were the most emphasized considerations of the interviewees in determining the sustainability of mHealth. The framework suggested that the 2 most important enabling resources for the successful implementation of mHealth interventions were access to mobile phones (or devices) and the availability of functioning stable telecommunication networks. Accordingly, the interviewees maintained and also suggested that mHealth interventions could be perceived as useful by both patients and health care providers if access to mobile phones, availability and affordability of the infrastructure for good quality (telecommunication) services, reduced burden of work for providers, the avoidance of system abuse, financial resources, and government and institutional support as well as legislation and policy support are assured (see Table 3).

Now we are having a lot of mobile phone services but we do have challenges with them. We need to have stable mobile phone services that are good. The services must be available everywhere.

Respondent: 3

When you are doing a project and you have somebody funding it like we did for the [mHealth] project, it’s cool. But then when the project comes to an end and the realities dawn on us, our governments should give money for some of these things.

Respondent: 1

It has to be a priority and all these things have to fit in the priorities of the Ministry of Health.

Respondent: 5

In addition, policy makers suggested that legislation, policies, and guidelines are needed to guide the activities of (health care) providers. However, they maintained that such policies for the explicit purposes of mHealth interventions should be appropriately informed by the evidence from, for example, pilot projects that first need to be conducted. Furthermore, they highlighted that the availability of financial resources would be an important enabling resource but that financial support and commitment from governments for mHealth interventions still remains low because of resource constraints.

[...] Yea, will you buy vaccines or you buy phones. I will rather buy vaccines than buy phones. Those are the realities that we deal with as people at the policy level. [...] So those are the trade-offs that we make at the national level and it's not an easy trade-offs [...] Also we need to really come out clearly what the parameters should be. We developed a mobile device guideline and we did advocate that the mobile phone is a medical device and so the health facilities have to provide them.

Respondent: 1

I believe in doing pilot projects before developing the policies because the findings of the pilot project should guide the policy. So, the immediate thing is to have a project with the NCD programme... it can be part of the priorities of the Ministry of Health.”

Respondent: 5

Interviewees identified several conditions that would enable patients to easily use mHealth interventions, including, for example, family support and availability of maintenance services. In the same vein, they emphasized that attention should be given to the suitability of the technologies for health care providers including certain specific features, such as the size of the screen.

...one mobile phone platform they created for health professionals to monitor those [patients] who are on medications, they secured an Android phone for all of them, I mean something with a bigger screen that they could do so many things on it. I think it has been tailored.

Respondent: 7

## Discussion

### Principal Findings

To our knowledge, this is the first study that has investigated the views of policy makers about factors that support successful implementation and scale-up of mHealth interventions. We found that policy makers in Ghana were well informed about the potential of using mobile phones for health promotion, prevention, and health service delivery—and they strongly supported the further expansion of mHealth in the country. The results of the study also showed that the mHealth PNE framework’s categories of *predisposing characteristics, needs, and enabling resources* are a useful guide for policy makers in ascertaining what critical factors would support the successful implementation of mHealth interventions. None of the policy makers stated any view that suggested that the framework has shortcomings. Rather, the responses of interviewed policy makers showed that they are thinking of many of the factors suggested by the mHealth PNE framework but that they tend to focus more on enabling resources than on predisposing characteristics and need. Finally, policy makers added several relevant factors under the categories of the mHealth PNE framework that should be considered when aiming to assure sustainability and scale-up of mHealth interventions.

These findings have several important implications for policy makers and researchers, as well as for the further refinement of the mHealth PNE framework. First, this study shows that policy makers are aware of many of the factors that have been described in the literature as particularly important for assuring successful implementation and scale-up of mHealth interventions for NCDs. For example, in line with previous literature [23], the participating policy makers highlighted that a positive attitude of both patients and providers toward mobile technologies is one of the most important factors influencing the perception of patients and providers that mHealth interventions are useful and easy to use. Similarly, their assessment that patients in need of special/urgent care are likely to benefit most from mHealth is in accordance with previous findings in the literature. This implies that policy makers in Ghana broadly agree with the findings of our systematic review [23] that it is important to consider context factors, that is, PNE, when developing and implementing mHealth interventions for NCDs. In fact, these context factors can be more important than the technical aspects of an intervention in determining its success [23,56-58].

Second, as the thinking of policy makers tends to focus on enabling resources, such as functioning telecommunication infrastructure, sustainable financing, and support from stakeholders, the mHealth PNE framework can be useful to facilitate a more holistic and systematic assessment of other factors supporting successful implementation, sustainability, and scale-up of mHealth interventions for NCDs. For example, future revisions of the Ghana eHealth Strategy [47] may benefit from considering the categories of the PNE framework to assure that new policies are developed, which will be adjusted to the needs of patients and providers, while taking into account their predisposing characteristics. The mHealth PNE framework (see Table 3) provides a long list of predisposing characteristics of patients and providers as well as of their needs, which can be used as a guide by policy makers during implementation, sustainability, and scale-up.

Third, this study has contributed to the refinement of the mHealth PNE framework by identifying additional patient- and provider-context factors that should be considered during implementation, sustainability, and scale-up of mHealth interventions. This includes patients’ predisposing characteristics, such as gender, urban/rural location, and personalization of technologies; patients’ need, such as their need for urgent/specialized care; and patients’ enabling resources, such as avoidance of abuse, partnership, and (family) support. Concerning providers, policy makers identified additional predisposing characteristics, such as good (provider-to-patient/community) relationships; additional need factors, such as the need for exchange of expertise and for continuity of care; and additional enabling resources, such as support from government and other stakeholders (see Table 3). Interestingly, policy makers noted that the increasing utilization of mobile phones by patients for services of other sectors, for example, in the financial/banking sector [59,60], may create a desire (or *need*) to also have mobile phone–based services in the health sector, which, in turn, may contribute to patients finding these technologies easy to use.

Finally, although the study shows that the categories of the framework are useful for policy makers, further (quantitative) research is required to test the validity of the framework and to explore the relative importance of the identified context factors for successful implementation, sustainability, and scale-up of mHealth interventions for NCDs. This may include, for example, studies testing the relevance of the identified context factors during the implementation of the WHO’s Package for Essential NCD Interventions (ie, integration of NCDs into primary health care) [9,61] using mobile technologies.

### Limitations

This study has several limitations. The recruitment of participants relied on the list of members of the technical working group for Ghana’s national NCD policy. This does not constitute a representative sampling of all relevant policy makers, and it has a bias toward the inclusion of policy makers with expertise in the area of NCDs, whereas possibly missing policy makers with expertise in the area of mHealth. However, the selected policy makers demonstrated that they had considerable knowledge in the area of mHealth, in addition to their long-standing experience from working in the health sector in Ghana.

The scope of this study was also limited by the use of qualitative methods. As a result, the contextual factors summarized in Table 3 are rather indicative. It is very likely that there are further predisposing characteristics, enabling resources, and need that are relevant for the implementation and scale-up of mHealth interventions for NCDs beyond those identified by the interviewed policy makers or by our systematic review [23]. In addition, the relative importance of the identified factors remains unknown. Therefore, more research is needed to confirm the mHealth PNE framework and to operationalize some of its categories. For example, concerning the interplay of predisposing characteristics and perceived usefulness (see Table 3), quantitative research is needed to confirm that a positive attitude toward mHealth is a predictor of perceived usefulness. This requires an operationalization for measuring a positive attitude and for quantifying its impact on the sustained use of mHealth for NCDs. Ideally, the mHealth PNE framework would be tested using a large dataset from a multicountry mHealth trial, allowing sufficient variation in the context factors that are hypothesized to influence long-term success of interventions.

### Conclusions

There is great potential for mHealth interventions to improve treatment and care for patients with NCDs in SSA. However, the views of policy makers about factors that support the successful implementation, sustainability, and scale-up of these interventions used to be unexplored. Our qualitative study found that policy makers in Ghana are aware of many of the factors that have been described in the literature as particularly important for assuring successful implementation, sustainability, and scale-up of mHealth interventions for NCDs. In addition, the study showed that the mHealth PNE framework is useful to guide policy makers toward a more holistic and systematic assessment of context factors that support intervention implementation, sustainability, and scale-up, such as predisposing characteristics of patients and providers, as well as their need. Furthermore, the study allowed to refine the mHealth PNE framework by identifying additional context factors under the categories of PNE that support implementation, sustainability, and scale-up of mHealth interventions for NCDs.

The implication of these findings is that policy makers may benefit from using the mHealth PNE framework at various stages of implementation and scale-up of mHealth interventions for NCDs. However, it is important to be aware that the framework is still in its early stages of development. Researchers may (and should) use the framework when investigating reasons for success (or failure) of interventions. Over the years, such an emerging body of evidence will contribute to confirming and/or refining the factors proposed by the mHealth PNE framework, and it may ultimately allow quantifying the relative importance of these factors.

## Appendix

Multimedia Appendix 1 Semistructured questionnaire.

## Abbreviations

eHealth: electronic health
mHealth: mobile health
NCD: noncommunicable disease
PNE: predisposing characteristics, need, and enabling resources
QCA: qualitative content analysis
SSA: sub-Saharan Africa
WHO: World Health Organization
