# Supplementary material for: Achieving Sustainability and Scale-Up of Mobile Health Noncommunicable Disease Interventions in Sub-Saharan Africa: Views of Policy Makers in Ghana
Source: JMIR Mhealth Uhealth. 2019 May 3;7(5):e11497. doi: 10.2196/11497 (PMC6524449; doi:10.2196/11497)
Supplement: Multimedia Appendix 1 [file mhealth_v7i5e11497_app1.pdf]

FEASIBILITY OF DEVELOPING AND IMPLEMENTING MOBILE PHONE-BASED NCD CARE IN GHANA

INTERVIEW GUIDE

PROFESSIONAL BACKGROUND

1. **1.1 Sex** female [ ] male [ ] other (specify) ___________________________

1.2 Age: 25 - 29 [ ] 30 - 34 [ ] 35 - 39 [ ] 40 - 44 [ ] 45 - 49 [ ] 50+ [ ]

1.3 Occupation/profession: ______________________________________

1.4 Current position ____

1.5 Years of working experience ______________

1.6 Can you describe your working experience with NCDs

NCD MANAGEMENT IN GHANA

2. What is your general assessment of how NCDs are managed?
   - **a.** Service provision
   - **b.** Access to (quality) care
   - **c.** Interventional activities
   - **d.** NCD care financing
   - **e.** Policies and guidance
3. Have there been major changes/reforms in NCD management in recent years?
4. What challenges confront NCD management?
5. What measures have been put in place to address these challenges?
   - **a.** (thinking about specific interventions, programmes or policy actions)
6. What is your assessment of the national policy for NCD management:
   - **a.** What has worked well?
   - **b.** What would need to be improved

MOBILE PHONE-BASED HEALTH INTERVENTIONS

7. Among these measures being taken, do any of them include the use of ICTs, particularly mobile phones?
   - **a.** If so, how?
   - **b.** If not, why?

8. Do you know of any of such interventions in other countries which is based on mobile phones for NCD care?
    a. If yes, kindly describe how the intervention(s) work?
9. In your opinion, how do you find these interventions suitable for the Ghanaian context?
10. What would be the factors to consider in implementing such interventions?
    a. Predisposing factors
    b. Enabling factors
    c. Need factors
    d. Usefulness
    e. Easy-to-use factors
11. For whom would these interventions work or not work, and why?
12. What impacts are they likely to exert on the health care system?
13. How could mHealth interventions be integrated into the existing health system?
14. What provisions would be required in the policy framework to guide the development and implementation of mHealth NCD care?
15. How could mHealth NCD Care be financed or what financial models would be appropriate for the implementation?
16. In addition to the issues discussed here, what other recommendations do you consider important for the improvement of NCD management as well as the health system?
17. Is there any issue to clarify or questions you would like ask?

THANK YOU VERY MUCH!
